# To evaluate the efficacy of Mobilization Techniques in Post-Traumatic stiff ankle with and without Paraffin Wax Bath

**DOI:** 10.12669/pjms.296.4127

**Published:** 2013

**Authors:** Sajid Rashid, Kamran Salick, Muhammah kashif, Awad Ahmad, Kashif Sarwar

**Affiliations:** 1 Dr. Sajid Rashid, BSPT, PP-DPT, HOD, Physiotherapy Department, The Children’s Hospital & the Institute of Child Health Multan, Multan, Pakistan.; 2 Prof. Dr. Kamran Salick, MD(USA), FCPS, Professor, Orthopedic Department, Nishtar Medical College & Hospital, Multan, Pakistan.; 3 Dr. Muhammad Kashif, FCPS, Associate Professor, The Children’s Hospital & the Institute of Child Health Multan, Multan, Pakistan.; 4 Dr. Awad Ahmad, Assistant Professor, Orthopedic Department, Nishtar Medical College & Hospital, Multan, Pakistan.; 5 Dr. Kashif Sarwar, BSPT, PP-DPT, Physiotherapist, Nishtar Medical College & Hospital, Multan, Pakistan.

**Keywords:** Mobilization techniques, Paraffin wax bath, Stiff ankle

## Abstract

***Objective: ***Mobilization techniques are frequently used by physiotherapists to reduce pain, improve joint movement and facilitate return to activities after injury. The objective of this study was to explore differences in the efficacy of Mobilization Techniques in Post-Traumatic stiff ankle with and without Paraffin Wax Bath.

***Methods: ***Thirty seven patients of Post Traumatic stiff ankle were recruited for the study at Sajid Physiotherapy & Rehabilitation center, Multan from March 2011 to February 2013. It was a randomized controlled trial and the patients with equal grades of severity were placed in control and study groups. Group A had nineteen patients and Group B had 18 patients. The inclusion criteria were age range from 20-60 years, pain, loss of ROM, with history of trauma and fracture of ankle. The patients with similar complaints but with surgical treatment were excluded. Group A was given mobilization techniques with paraffin wax bath while group B was treated without paraffin wax bath. Improvement was observed by EscolaPaulista de Medicina Range of Motion (EPM-ROM) scale and visual analogue scale (VAS). After ten weeks of treatment, the patients were re-evaluated by an orthopedic surgeon and a Physiotherapist for their symptoms and ROM. t-test was applied to compare outcome between two groups and p < 0.05 was considered to be statistically significant.

***Results: ***Group A had nineteen patients and Group B had 18 patients and both were treated for ten weeks. There were 12 male and 7 female patients in group A and 10 male and 8 female in group B. At the start of treatment the basic characteristic were similar in both the groups. Deficits in dorsiflexion, planterflexion, inversion, eversion pain and stiffness were measured before and after the treatment period. Pain relief was found better in both groups which were considered statistically significant with p=0.001, group A (1.135 ± 0.359) vs. group B (1.135 ± 0.359). ROM in pre and post treatment degrees showed that dorsiflexion was significantly increased in group A (1.135 ± 0.359) vs. group B (1.135 ± 0.0359).) and planterflexion was in group A (1.337 ± 0.422) vs. group B (0.841 ± 0.264). Functional movement showed improvement in inversion in group A (0.875 ± 0.276) vs. group B (0.966 ± 0.305) and in eversion in group A (0.948 ± 0.300) vs. group B (0.674 ± 0.213). Mobilization Techniques followed by wax bath resulted in significant improvements of range of motion (ROM), clinical and functional changes. Wax bath alone had no significant effect. After ten weeks intervention treatment, t-test was applied to compare outcome between the two groups and p=0.001to 0.004 in group A and p= 0.104 to 0.168 in group B, (p<0.05) was obtained which shows statistical significance.

***Conclusion: ***Joint mobilization and wax bath therapy is an effective and beneficial tool to improve the symptoms and quality of life in post traumatic stiff ankle patients. Joint mobilization techniques combined with wax bath are more effective in the management of post-traumatic stiff ankle as compared to wax therapy alone.

## INTRODUCTION

The ankle is particularly vulnerable to trauma. The bones of the ankle are subcutaneous. The soft tissue envelope consists of only skin, tendon, and neurovascular structures anterior, lateral, and medial to the joint. Only in the posterior quadrant is there a modest muscular envelope. In addition, the ankle joint does not tolerate deformity or articular incongruity after trauma. Signs of a fracture include immediate and severe pain, swelling, bruising, tenderness to the touch, inability to put weight on the foot and visual deformity.

The post-traumatic stiff ankle generally leads to disuse of foot function, due to restricted range of motion and loss of muscle strength. The physical therapists rehabilitate the patients with post-traumatic stiff ankle by joint mobilization techniques, stretching and strengthening exercises. If the patients are not rehabilitated, they will develop contractures and foot goes to valgus deformity & will result in a position of dysfunction.^2^ The physical therapy plan of care is based on physical examination, includes evaluation of ROM, muscle strength, edema, gross sensation, bone healing, and adhesions. The key principle of ankle fracture rehabilitation is to maintain the restored anatomy of the ankle joint, while restoring full range of ankle motion as early as possible.^3^Walking as soon as the treating doctor indicates that it is safe to do so using a Removable Plastic Cast Walker is also important. This prevents complications such as muscle wastage, joint stiffness and degeneration of joint cartilage. By putting a carefully controlled load through the injured ankle it also stimulates fracture healing and helps to prevent non union.

The improvement in joint ROM is the key component of physical therapy management, due to musculotendinous tightness. Joint mobilization or manipulation involves passively moving the joints of the human body in a way that cannot be accomplished by active movement (movements you perform yourself). It involves small sliding movements of the bone surfaces into the direction of restriction. Translations, glides even rolling motions are assessed and corrected in during a typical joint mobilization treatment.^4^There are several different grades of joint mobilization ranging from grade one to five. Grades one, two and three are gentle movements designed to prevent adhesion and reduce pain. Grade four mobilizations, the most commonly performed mobilization is a stretching motion performed at the joint’s end range. Grade five mobilizations is designed to restore mobility to the joint, also have the added benefit of pain reduction due to a restoration of normal joint arthrokinematics (glide, roll etc.).

The joint mobilization techniques are used to improve joint ROM, by producing passive glides with distraction between the articular surfaces of hand joints to manage pain, break adhesions, and improve joint ROM.^5^ There are many clinical reports concerning the use of paraffin wax bath for various types of conditions including post traumatic stiff joints.^6^ The wax softens the dry skin making it supple and heat increases blood flow through skin. Wax therapy also stimulates the sweat gland, especially those which retain their nerve supply.^7^The paraffin wax bath is commonly used as effective remedy to improve circulation and promotes relaxation. Hands and feet are most common segments to be treated with paraffin wax bath in physical therapy.^8^

## METHODS

Diagnosed cases of post traumatic stiff ankle presenting at Sajid Physiotherapy & Rehabilitation center, Multan from March 2011 to February 2013, were assessed by an orthopedic surgeons. This study was a randomized controlled trial and thirty seven diagnosed cases were recruited and divided in a control and study group. Group A had nineteen patients and Group B had 18 patients. The inclusion criteria were age range from 20-60 years, pain, loss of ROM, with history of trauma and fracture of ankle. The patients with similar complaints but with surgical treatment were excluded. Guardians and patients were informed about the benefits/risks of the management plans and informed consent was taken. Study protocol was approved by the Institutional Ethical Committee.

All the information was recorded and study variables were measured at the base line including age, gender, foot involved, pain intensity score, dorsiflexion, planterflexiom, inversion and eversion. The ROM was measured by goniometer in supine position.^9^

Both groups were called on daily basis except Sundays, Group A patients were treated with joint mobilization techniques and paraffin wax bath. While Group B patients were treated with only joint mobilization techniques.

Wax therapy was given with paraffin wax bath machine by Enraf-Nonius, Netherlands. Paraffin wax is solid at room temperature and begins to melt above 37 C(99F) and melting point is 46 to 68 C (115 to 154F). The paraffin wax bath was applied 25 minutes prior to joint mobilization techniques including glides of the articular surface in supine position. The joint mobilization grade -1 and grade 2 were usedfor pain management and relaxation, while Grade 3 were Used to stretch joint structures and increase joint play.

The study variables were calculated at the completion of 10 weeks physical therapy treatment program. All the patients were re-evaluated (by panel of Orthopaedic surgeons and physiotherapist) for their pain intensity score, dorsiflexion, planterflexiom, inversion and eversion on EscolaPaulista de Medicina Range of Motion (EPM-ROM) scale and visual analogue scale(VAS) 0/10. Statistical analysis was done by SPSS version-20 and t-test was applied to compare outcome between two groups and p<0.05 was considered to be statistically significant.

## RESULTS

Age of the patient was 20 -56 years in group A and in group B 21-62 years. Mean age of the patients was 36.43 ± 0.8 in group A and in group B was 41.33 ± .07 years. Thirty seven patients with post traumatic stiff ankle were recruited and randomly divided in a control and study group. Group A had nineteen patients and Group B had 18 patients and treated for ten weeks. There were 12 male and 7 female patients in group A and 10 male and 8 female in group B. At the start of treatment the basic characteristic were similar in both the groups. Deficits in dorsiflexion, planterflexion, inversion, eversion pain and stiffness were measured before and after the treatment period. The control group was measured at corresponding times. Pain relief was found better in both groups which were considered statistically significant with p=0.001, group A (1.135 ± 0.359) vs. group B (1.135 ± 0.359). ROM in pre and post treatment degrees showed that dorsiflexion was significantly increased in group A (1.135 ± 0.359) vs. group B (1.135 ± 0.0359).) and planterflexion was in group A (1.337 ± 0.422) vs. group B (0.841 ± 0.264). Functional movement showed improvement in inversion in group A (0.875 ± 0.276) vs. group B (0.966 ± 0.305) and in eversion in group A (0.948 ± 0.300) vs. group B (0.674 ± 0.213). Mobilization Techniques followed by wax bath resulted in significant improvements of range of motion (ROM), clinical and functional changes. Wax bath alone had no significant effect. After ten weeks intervention treatment, t-test was applied to compare outcome between the two groups and p=0.001to 0.004 in group A and p= 0.104 to 0.168 in group B, (p<0.05) was obtained which shows statistical significance. Table-II.

**Table-I:** Descriptive statistics

*Characteristic *	*Group A*	*Group B*
Male	12(63.15)	10(55.55)
Female	7(36.84)	8(44.44)
Rt. Foot Involvement	11(57.89)	14(77.77)
Lt. Foot Involvement	8(42.10)	4(22.22)
Disability	9(47.36)	7(38.88)
Active Life	10(52.63)	11(61.11)

Group A= Patients treated with mobilization techniques and paraffin wax bath.

Group B= Patients treated without paraffin wax bath.

**Table-II:** Clinical and functional changes after 10 weeks wax bath treatment

*Characteristic*	*Group A*		*Group B*	
	*Std.deviation*	*p-value*	*Std. deviation*	*p-value*
Pain score	1.135 ± 0.359	0.001	1.135 ± 0.359	0.001
Dorsiflexion	0.816 ±0.258	0.004	0.843 ± 0.266	0.161
Planterflexion	1.337 ± 0.422	0.013	0.841 ± 0.264	0.151
Inversion	0.875 ± 0.276	0.003	0.966 ± 0.305	0.081
Eversion	0.948 ± 0.300	0.002	0.674 ± 0.213	0.193

Group A= Patients treated with mobilization techniques and paraffin wax bath.

Group B= Patients treated without paraffin wax bath.

## DISCUSSION

 Over many decades mobilization techniques were used for increase of joint range but now a days paraffin wax bath is also being used as alternative treatment in post traumatic stiff joints.^4^ Various reports have been published, about mobilization techniques with paraffin wax bath as a new treatment option in the management of post traumatic stiff joints^10^The overall results of this study showed the improved ankle function and immediate pain relief in group A.

Studies conclude that paraffin bath therapy is not effective when used alone (i.e. when not paired with therapeutic exercise). One such study was done, to create guidelines for thermotherapy interventions for adult patients with RA, by Members OP et al. they found no statistically significant difference for patients who had wax applied to the hand and ankle versus a control after one month.^10^ It was, however, shown that wax combined with exercise versus a control has proved clinically important benefits. Results from our study showed an overall improvement in group A, treated with mobilization techniques and wax bath. In this study we used EscolaPaulista de Medicina Range of Motion (EPM-ROM) scale and visual analogue scale (VAS). Scoring system to see the improvement of the patients. Wax therapy and mobilizations techniques were being used as a tool of treatment in arthritis patients in previous studies by various authors.^11^All these studies supported that mobilization techniques and Wax bath is a useful remedy in stiff joints, which is in accordance with our study.

BeritDellhag et al. used wax bath and exercises on 52 patients.^12^Both hands dipped five times into a 47 to 50 C, then wrapped in paper, in which they were kept for 20 minutes. Patients in that study had significant improvement, similar to present outcome, in which we used wax bath for 25 minutes daily prior to joint mobilization techniques including glides of the articular surface of joint.

As regards the mechanism by which Wax is working , Pain is a way for the body to indicate that something is wrong and while the body releases endorphins to the site of the pain for relief, the pain will persist if the path is blocked and the endorphins cannot get through. Endorphins travel through the blood, airway, and the sub-cutaneous layer — a watery layer between the muscle tissue and skin. The sub-cutaneous layer is accessible through thousands of tiny holes in the skin. Slowly raising the temperature of the skin around the affected area warms the sub-cutaneous layer and triggers a gland at the base of the brain to release endorphins to the site of the pain, thereby bringing relief. Wax may also effect and stimulating to release endorphins.^8^

In the research review Thermotherapy for treating Rheumatoid Arthritis by Welch V. et al., researchers looked at different types of thermotherapy and their effectiveness for treating rheumatoid arthritis.^13^ Interventions other than paraffin were hot pack, cold pack, faradic bath, and therapeutic ultrasound;they found statistically significant results favoring paraffin wax combined with exercise.

O’Brien and Vicenzino used a mobilization technique in stiff ankle.^4^The intervention included a sustained posterior glide to the distal fibula while the patient actively inverted the ankle. The suggested benefits of treatment included reduced pain and increased ROM. They concluded mobilization exercise and splinting can prevent contractures in stiff ankle.

Green and colleagues used a parallel-design randomized controlled trial to compare the effect of joint mobilization in addition to rest, ice, compression and elevation (RICE).^14^They concluded on the basis of available studies of joint mobilization techniques, which are effective for pain management and improve function.The results from the present study support an overall improvement in pain, dorsiflexion, planterflexiom, inversion and eversion.

## CONCLUSION

Joint mobilization and wax bath therapy is an effective and beneficial tool to improve the symptoms and quality of life as compared to wax therapy alone. The joint mobilization and wax bath intervention successfully enhanced patient-oriented and clinician-oriented measures of function.

## Notes

***Grant Support & Financial Disclosures: ***None
